# Characterization of oat chromosome rearrangements in advanced breeding lines and implications for genetic improvement

**DOI:** 10.1007/s00122-026-05332-4

**Published:** 2026-08-06

**Authors:** Guilherme Oliveira, Wubishet Bekele, Françoise Dalprá Dariva, Sumandeep Bazzer, Jason Fiedler, Raja Sekhar Nandety, Jean-Luc Jannink, Melanie Caffe

**Affiliations:** 1 Department of Agronomy, Horticulture, and Plant Science, South Dakota State University (SDSU), Brookings, SD USA; 2 Ottawa Research and Development Centre, Agriculture and Agri-Food Canada, Ottawa, ON Canada; 3 USDA-ARS Cereal Crops Improvement Research Unit, Edward T. Schafer Agricultural Research Center, Fargo, ND USA; 4 R.W. Holley Center for Agriculture and Health, USDA-ARS, Ithaca, NY USA

## Abstract

Large-scale chromosomal rearrangements or structural variants (SVs) have shaped the evolution of *Avena* species, driving domestication and adaptation in oats (*Avena sativa* L.). The legacy of these reorganizations, including translocations and inversions, remains evident, yet their frequency and impact on phenotypic and genotypic variability in oat breeding populations remain unclear. The objective of this study was to characterize chromosome rearrangements in 1230 oat breeding lines using genotyping-by-sequencing and to utilize this information in genomic loci discovery and prediction. Principal component analysis (PCA) based on single-nucleotide polymorphisms (SNPs) identified distinct clusters at the known locations of 1A/1C translocation, 3C inversion, 4C inversion, and 7D inversion. The distribution of the clusters of the four rearrangements in the genome-wide diversity space of our panel confirmed the role of SVs in population structure, with 1A/1C showing the most significant divergence. Suppression of recombination due to SVs was shown by higher linkage disequilibrium (LD) observed in all samples compared to lines within individual SV clusters. Genome-wide association studies (GWAS) identified markers for grain yield and agronomic traits, with some differences among cluster-based populations. Genomic prediction accuracies improved when cross-validation was performed within SV clusters rather than across the entire dataset. However, using SV clusters as covariates did not improve GWAS and prediction accuracies. These results suggest that SVs cause population stratification, reduce recombination rates, hinder gene discovery, and affect genomic prediction accuracy. Characterizing oat germplasm for SVs can improve breeding decisions, from optimizing parental selection to enhancing genomic predictions, accelerating genetic gains for key traits.

**Supplementary Information:**

The online version contains supplementary material available at 10.1007/s00122-026-05332-4.

## Introduction

Oats (*Avena sativa* L.), the world's seventh-largest crop by production (FAOSTAT 2021), is an allohexaploid (2*n* = 6*x* = AACCDD) with a large and intricate genome (Peng et al. 2022). The latest genome assembly (OT3098 v2) is approximately 10.8 gigabases (Gb) in size (https://www.ncbi.nlm.nih.gov/datasets/genome/GCA_916181665.1/, accessed April 2025) and contains a high proportion of repetitive sequences (Liu et al. 2019). In addition to the large-size and repeat-rich genome, a recent study analyzing cultivated oat (2*n* = 6x = AACCDD) and its close relatives, *Avena longiglumis* (2*n* = 2*x* = AA) and *Avena insularis* (2*n* = 4*x* = CCDD), uncovered extensive genomic rearrangements and structural variants throughout the polyploidization history of this species, resulting in a mosaic-like genome architecture (Kamal et al. 2022). Among the main characteristics identified are the inconsistent increase or decrease in gene number and recombination rates with increasing distance from the centromere, the loss of large genomic regions from the C to the A and D subgenomes, and crossover events between homoeologous chromosomes (Kamal et al. 2022). Such a complex genome has led to pseudo-linkage and reduced recombination, which negatively influences inheritance patterns, mapping accuracy, and the utilization of quantitative trait loci (QTLs) (Tinker et al. 2022). Consequently, genome structure and patterns are among the primary opportunities and barriers to genetic gains for several traits in oats. It is an opportunity when clusters of desirable genes remain intact for generations.

Historical breeding efforts to introduce resistance genes for crown rust (*Puccinia coronata* f. sp. *avenae*) in oats from alien species (Park et al. 2022) may have exacerbated the genome structural and genetic complexity of modern oat breeding material and could potentially impair genetic gain. Most of the cataloged and exploited resistance genes in oat breeding originate from two other hexaploid *Avena* species, *Avena byzantina* (2*n* = 6*x* = AACCDD) and *Avena sterilis* (2*n* = 6*x* = AACCDD), which, when crossed with oats, produce fertile progeny (Park et al. 2022). While these genes provided effective resistance at the time of their introgression, their resistance has since broken down; however, the associated introgressions and chromosomal rearrangements from these species remain present in recently developed oat breeding populations (Bekele et al. 2025). Many also attribute these introductions for the greater diversity in North American breeding programs compared to European breeding germplasm. These rearrangements also contribute to pseudo-linkage and reduced recombination in oats. Furthermore, the exploration of alien species as sources of crown rust resistance was not restricted to hexaploid *Avena* species. The development of synthetic hexaploid to incorporate resistance from diploid and tetraploid species of the *Avena* genus was also widely tested and utilized (Rines et al. 2007, 2018). A famous case of a synthetic hexaploid used in oat breeding targeting crown rust resistance is the ‘Amagalon’ line, a cross between *Avena longiglumis* (2*n* = 2x = AA) and *Avena magna* (2*n* = 4*x* = CCDD) (Park et al. 2022). The effects and frequency of using Amagalon or its derived breeding lines (e.g., cultivar HiFi, McMullen et al. 2005) as parents in the crossing blocks have been associated with greater genetic differences among oat breeding programs, primarily due to the haplotypes related to chromosomal rearrangements inherited from Amagalon (Bekele et al. 2025).

Given the challenges associated with the mosaic-like genome structure and the historical usage of wild species in oat breeding, understanding *Avena* species genomes and characterizing chromosome rearrangements is a fundamental research area for genetic improvement in this crop (Tinker et al. 2022; Tomaszewska et al. 2022). In this context, a pangenome study (Avni et al. 2025) and a population structure study to understand the relationships between oats and wild species (Bekele et al. 2025) were published, clarifying the origin, presence, and location of several important chromosome rearrangements. Among the rearrangements found in these two studies, a translocation from chromosome 1C to 1A, an inversion on chromosome 3C, an inversion on chromosome 4C, and an inversion on chromosome 7D received extra attention due to their potential roles during domestication and local adaptation (Jellen and Beard 2000; Wooten et al. 2007; Santos et al. 2006; Peng et al. 2022; Tinker et al. 2022). For instance, the inversion on chromosome 7D was associated with heading date differences in diverse oat lines (Avni et al. 2025).

Despite these important findings, it remains unclear how these chromosome rearrangements affect key traits and how an oat breeder should manage them and their associated lack of recombination, during the breeding process. Specifically, it is necessary to understand how those rearrangements may affect results from genome-wide association study (GWAS) and from genomic prediction models. These analyses typically do not account for such rearrangements. GWAS models usually control population structure and relatedness to avoid false positive associations (Tibbs Cortes et al. 2021). However, correcting for population structure in GWAS can partially mask the genetic variation underlying the trait of interest (Bernardo 2020), particularly when the true biological signals are correlated with the population structure (Tibbs Cortes et al. 2021). To date, no studies have compared GWAS results in subpopulations based on known chromosomal rearrangements, and the entire population. Similarly, genomic selection (GS), a genomics-assisted breeding tool that exploits genome-wide markers to predict breeding values, has become a common approach in several plant breeding programs (Alemu et al. 2024). Among the factors that influence prediction accuracies is the relatedness between the training and testing populations, since predictions are made based on the covariances between the lines between both sets (Clark et al. 2012; Hickey et al. 2014). In this sense, understanding the population structure potentially caused by chromosomal rearrangements could be crucial for optimizing training populations, as the presence of subpopulations within the training population negatively affects prediction accuracies (Asoro et al. 2011).

Therefore, the objectives of this study are to characterize oat breeding lines for the major chromosomal rearrangements in oats (1A/1C translocation, 3C inversion, 4C inversion, and 7D inversion), to explore their effect on major agronomic traits of oats and to determine their impact on the identification of genomic regions associated with important oat breeding traits through GWAS, as well as on the accuracy of genomic predictions.

## Materials and methods

### Plant materials and phenotyping

This research utilized a set of 1230 lines, comprising 12 oat cultivars and 1218 breeding lines, evaluated in Preliminary Yield Trials (PYTs) conducted in 2015, 2016, 2017, 2020, 2021, and 2022 by the South Dakota State University (SDSU) Oat Breeding Program. From the 1230 PYT lines for this study, 217 were evaluated in 2015, 133 in 2016, 211 in 2017, 220 in 2020, 215 in 2021, and 222 in 2022, with only lines used as checks overlapping in minimal cases across years. A detailed description of these nurseries is provided in Bazzer et al. (2025) and Oliveira et al. (2025).

Subsequent analyses focused on four traits: crown rust severity (CRS), grain yield (GYL), heading date (HDT), and plant height (PHT) (Avni et al. 2025; Bekele et al. 2025). CRS was visually assessed by estimating the percentage of leaves covered with pustules in the plants within the plots, GYL was recorded in kilogram per hectare at 14% moisture, HDT was recorded as the calendar day on which 50% of the panicles had emerged from the main stem in each plot, and PHT was measured as the distance in centimeters from the soil surface to the tip of the tallest panicle.

### Genotyping

The set of 1230 breeding lines was genotyped using a genotyping-by-sequencing (GBS) approach at the USDA-ARS Genotyping Lab (Fargo, North Dakota), as described in Bazzer et al. (2025) and Oliveira et al. (2025). Following a protocol adapted from Poland et al. (2012), GBS libraries were prepared using the restriction enzymes *PstI* and *MspI*, and sequenced on an Illumina NextSeq 2000 system with 150 bp paired end reads (Illumina, San Diego, USA). Sequence data were quality filtered and adapter-trimmed using *bbduk* (BBTools, https://jgi.doe.gov/data-and-tools/bbtools), then aligned to the oat reference genome, *Avena sativa OT3098v2* (https://wheat.pw.usda.gov/jb/?data=/ggds/oat-ot3098v2-pepsico) using Bowtie2 (Langmead and Salzberg 2012). Aligned reads were sorted and indexed with SAMtools (Danecek et al. 2011, 2021), and variants were called with BCFtools (Danecek et al. 2021) using a read mapping quality threshold of ≥30. VCF files were filtered using VCFtools with max-missing 50%, MAF ≥ 0.01, and minimum depth ≥4. A total of 38,070 SNP markers comprised the initial genotypic data. Missing genotype calls were imputed using the LD-kNNi method from TASSEL v 5.0 (Bradbury et al. 2007). Posteriorly, lines and SNPs were filtered using the following criteria: (1) lines that had more than 60% of SNPs data missing, (2) lines with heterozygosity proportion higher than 15%, (3) SNPs with heterozygosity higher than 5%, and (4) SNPs with MAF lower than 5%. After the filtering process, a total of 9341 SNP markers were retained. Flanking sequences (100 bp) were extracted from around each SNP with BEDtools (Quinlan and Hall 2010) and used as query for BLASTn alignment to the Sang v1 genome sequence (karyotype/pseudo-molecule: presence of 1A/1C translocation and 7D inversion; 3C-inverted and 4C-non-inverted). The lifted-over SNP position and strand in the new genome was identified from the top alignment with a custom awk script.

### Population structure and linkage disequilibrium analyses

Here we specifically investigated four structural variations (SVs): 1A/1C translocation (genomic location: Chromosome 1A, 420–525 Mbp), the 3C inversion (genomic location: Chromosome 3C, 80–404 Mbp), the 4C inversion (genomic location: Chromosome 4C, 208–497 Mbp), and the 7D inversion (genomic location: Chromosome 7D, 256–412 Mbp) (Bekele et al. 2025) in our set of 1230 breeding lines, as well as to assess the recombination rates within these regions, population structure and LD analyses were performed. Initially, the SNPs inside the SVs were filtered using the squared correlation coefficient (*r*^2^) between SNPs, with a threshold of 0.2, though the SNPrelate R package (Zheng et al. 2012), to avoid strong influence of subclusters due to high linkage disequilibrium between SNPs. Subsequently, using the filtered set of markers for each chromosome rearrangement region (Supplementary Table 1), k-means clustering was applied, through the k-means function from the stats R package, to identify distinct clusters within these regions. Clusters were identified using the minimum, maximum, and median values of the first principal component. To determine the optimum cluster number, the ratio of between-cluster to total sum of squares was calculated and the smallest number of clusters that produced a ratio greater than 0.9 was chosen. Subsequently, the clusters were characterized by PCA estimation using the base R function prcomp and the filtered SNPs from each region. The variance explained by the first ten principal components were shown through bar plots, and the first two principal components were visualized through scatterplots. A PCA was performed using the full marker matrix (9341 SNPs) to investigate the relationships among clusters and assess the effects of chromosomal rearrangements and population structure based on genome-wide information. Additionally, the loadings (eigenvector coefficients) for markers on PC1 and PC2 were examined to confirm whether the visual patterns reflected potential relationships between population structure and the chromosomal rearrangements. Frequency bar graphs were also developed to examine cluster proportions across nurseries. Pairwise LD was estimated for the chromosomes with rearrangements (1A, 3C, 4C, and 7D) using squared allele correlation (*r*^2^), as implemented in the LDcorSV R package (Mangin et al. 2012). Based on the LD analysis, genetic diversity within these chromosomes was further investigated using progenies developed from parents belonging to different genomic clusters. These parents, as well as their progenies, are included in the set of 1230 lines analyzed in this study. Additionally, to confirm the genetic differences among the identified clusters, the marker variants and their distribution across the whole chromosome and the chromosome rearrangement regions were examined using the geom_raster function from the ggplot2 R package (Wickham 2016). Pairwise genetic distances were estimated using basic R functions to assess variability within clusters, and significance was tested through nonparametric tests.

### Phenotypic data analysis

Adjusted means were calculated using the best linear unbiased estimates (BLUEs), providing accurate phenotypic values for the subsequent analysis. The multi-step pipeline to estimate BLUEs for each genotype across multiple environments and traits follows. First, for each environment–trait combination, we fitted a spatial model using the SpATS R package (Rodríguez-Álvarez et al. 2018) to account for within-field spatial variability (Eq. 1):

1 $$y_{ijk} = u + G_{i} + f\left( {\mathrm{row,col}} \right) + R_{j} + C_{k} + e_{ijk}$$

In Eq. 1, ${y}_{ijk}$ is the trait value; u is the overall mean; ${G}_{i}$ is the fixed effect of genotypes i; $f\left(\mathrm{r}\mathrm{o}\mathrm{w},\mathrm{c}\mathrm{o}\mathrm{l}\right)$ is the spatial trend modeled by a two-dimensional P-spline surface; ${R}_{j}$ and ${C}_{k}$ are random effects for row and column, respectively; and ${e}_{ijk}$ is the residual error. Second, these BLUEs value were compiled, targeting standardized genotype responses across environments and traits and cross-environment modeling. Third, to estimate genotype performance adjusted across environments, a linear mixed model (Eq. 2) using the lme4 R package was applied for each trait (Bates et al. 2015).

2 $$y_{ij} = u + G_{i} + E_{j} + e_{ijk}$$

In Eq. 2, ${y}_{ij}$ is the BLUE from the first step for genotype i in environment j; ${G}_{i}$ is the fixed effect of genotype; ${E}_{j}$ is the random effect of environment: ${E}_{j }\sim {\rm N}(0,{\sigma}_{E}^{2})$; and ${e}_{ijk}$ is the residual error: ${e}_{ijk}\sim {\rm N}(0,{\sigma }^{2})$.

From the BLUEs obtained through Eq. 1, linear mixed-effects models were initially conducted with each trait as the response variable, including the main effects of the four structural variations and their interactions with PYT. Line was treated as a random effect to account for genetic background and repeated measures. Based on this model, analysis of variance (ANOVA) was performed to test the significance of fixed effects and their interactions, and Šidák corrections were applied for multiple comparisons. In addition, a linear model with cluster as fixed effects and post hoc test (Tukey’s test) were used to assess the effects of clusters on phenotypes across PYTs. The frequency distributions of CRS, GYL, HDT, and PHT BLUEs were visualized with boxplots generated using the R package ggplot2 (Wickham 2016) grouped by clusters. From the BLUEs obtained through Eq. 2, boxplots were also created to evaluate data range and distribution shifts in the cluster-based population compared with the whole population. These BLUEs were subsequently used for genome-wide association studies and genomic prediction.

### Genome-wide association studies

GWAS analyses were performed to identify significant associations between the 9341 SNP markers and the traits analyzed across the whole population, both with and without chromosomal rearrangements included as covariates, as well as within subpopulations defined by chromosomal rearrangement clusters. The associations were performed in the GAPIT R package (Wang and Zhang 2021). Among the model options provided by GAPIT, the 'BLINK' (Bayesian information and linkage disequilibrium Iteratively Nested Keyway) model was chosen due to its effectiveness in minimizing false positives and identifying more true positive associations compared to other models (Huang et al. 2019). In the BLINK methodology, two fixed effect models (FEMs) (Eqs. 3 and 4) and one filtering process are conducted (Eq. 5).

3 $$y_{i} = S_{i1}^{*} b_{1} + S_{i2}^{*} b_{2} + \cdots + S_{ik}^{*} b_{k} + S_{ij} d_{j} + e_{i}$$

4 $$y_{i} = S_{i1}^{*} b_{1} + S_{i2}^{*} b_{2} + \cdots + S_{ik}^{*} b_{k} + e_{i}$$

5 $${\mathrm{BIC}} = - 2{\mathrm{LL}} + 2k{\mathrm{Ln}} \left( n \right)$$

In the first FEM (Eq. 3), the markers are tested one at a time, with pseudo-quantitative trait nucleotides (QTNs) added as covariates to improve the model and reduce false positives. As a result of the first FEM,* p*-values for all the markers are obtained. Based on the* p*-values and correlation between the markers, the second FEM (Eq. 4) optimizes the QTN selection. In Eqs. 3 and 4, ${y}_{i}$ is the observation of the *i*th individual; ${S}_{ik}$ represents the genotypes of k pseudo-QTNs; ${S}_{ij}$ represents genotype of the ith individual and jth genetic marker; ${b}_{k}$ represents the effects of the k pseudo-QTNs; ${d}_{j}$ represents the effect of the jth genetic marker; and ${e}_{i}$ represents the residuals (Huang et al. 2019). The optimization (Eq. 5) uses Bayesian information criteria (BIC), where $\mathrm{L}\mathrm{L}$ is the log likelihood; k represents the number of pseudo-QTNs; $\mathrm{L}\mathrm{n}$ is the natural log; and n is the number of individuals (Huang et al. 2019). The process described is repeated several times until the QTNs selected are the same across the repetitions. The selected threshold was determined using the Bonferroni correction method, corresponding to* α* = 0.05, serving as the significance threshold for identifying and classifying SNP markers associated with traits.

### Genomic predictions

To explore whether the chromosome rearrangement clusters identified through population structure analysis influence genomic prediction models for CRS, GYL, HDT, and PHT, multiple cross-validation scenarios were tested. In the first scenario, the entire dataset was used (ALL; *n* = 1230). The remaining scenarios were defined based on the cluster groups associated with chromosome rearrangements, resulting in nine additional models: 1A/1C cluster 1 (*n* = 949); 1A/1C cluster 2 (*n* = 281); 3C cluster 1 (*n* = 342); 3C cluster 2 (*n* = 888); 4C cluster 1 (*n* = 1113); 4C cluster 2 (*n*= 117); 7D cluster 1 (*n* = 865); 7D cluster 2 (*n* = 158); and 7D cluster 3 (*n* = 207). In each scenario, the respective datasets were randomly partitioned into five folds (80% training, 20% testing), ensuring approximately equal numbers of oat genotypes per fold. Prediction accuracy was evaluated using the Pearson correlation coefficient (*r*) between the multi-step BLUEs and the predicted phenotypes based on estimated genetic values. The mean accuracy was calculated by averaging the correlation across all five folds. This entire cross-validation process was replicated 100 times using different random partitions. Thus, the final accuracy for each genomic prediction scenario was the mean of these 100 replications. The GBLUP model, a linear mixed model that differentiates between random and fixed effects (Eq. 6), was used to assess the ten genomic prediction scenarios.

6 $$y = {{\rm X}}\beta + {{\rm Z}}u + e$$

In Eq. (6), y represents the vector of phenotypic observations; ${\rm X}$ is the full-rank design matrix for the fixed effects β; ${\rm Z}$ is the design matrix for the random effects u, with $u \sim {\rm N}(G{\sigma }^{2} \mu )$, where G is the genomic relationship matrix calculated using all available molecular markers (Endelman 2011). In addition, if the cross-validation with the highest prediction accuracy for a specific trait was not the model using the whole set of individuals, meaning that the highest accuracy was achieved for lines within a specific cluster, a secondary cross-validation was performed using the entire dataset, this time including that cluster as a covariate in the GBLUP model.

## Results

### Local and genome-wide genetic structure of advanced breeding lines

**Fig. 1:**
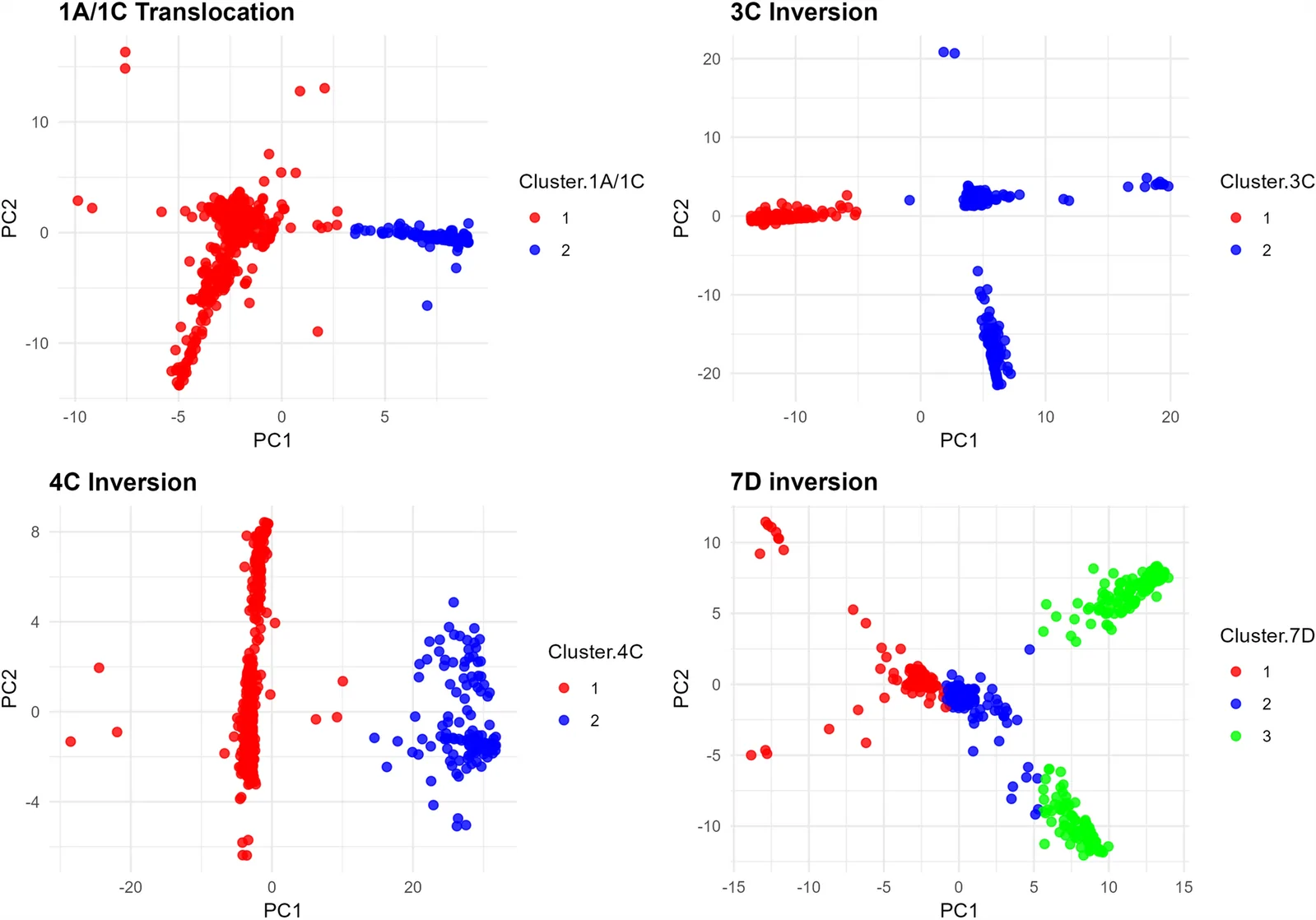
Distribution of 1230 oat breeding lines along the first two principal components based only on genomic information from chromosomal rearrangements signature markers. The genomic position and number of the markers used in each graph are described on the Supplementary Table 1

**Fig. 2:**
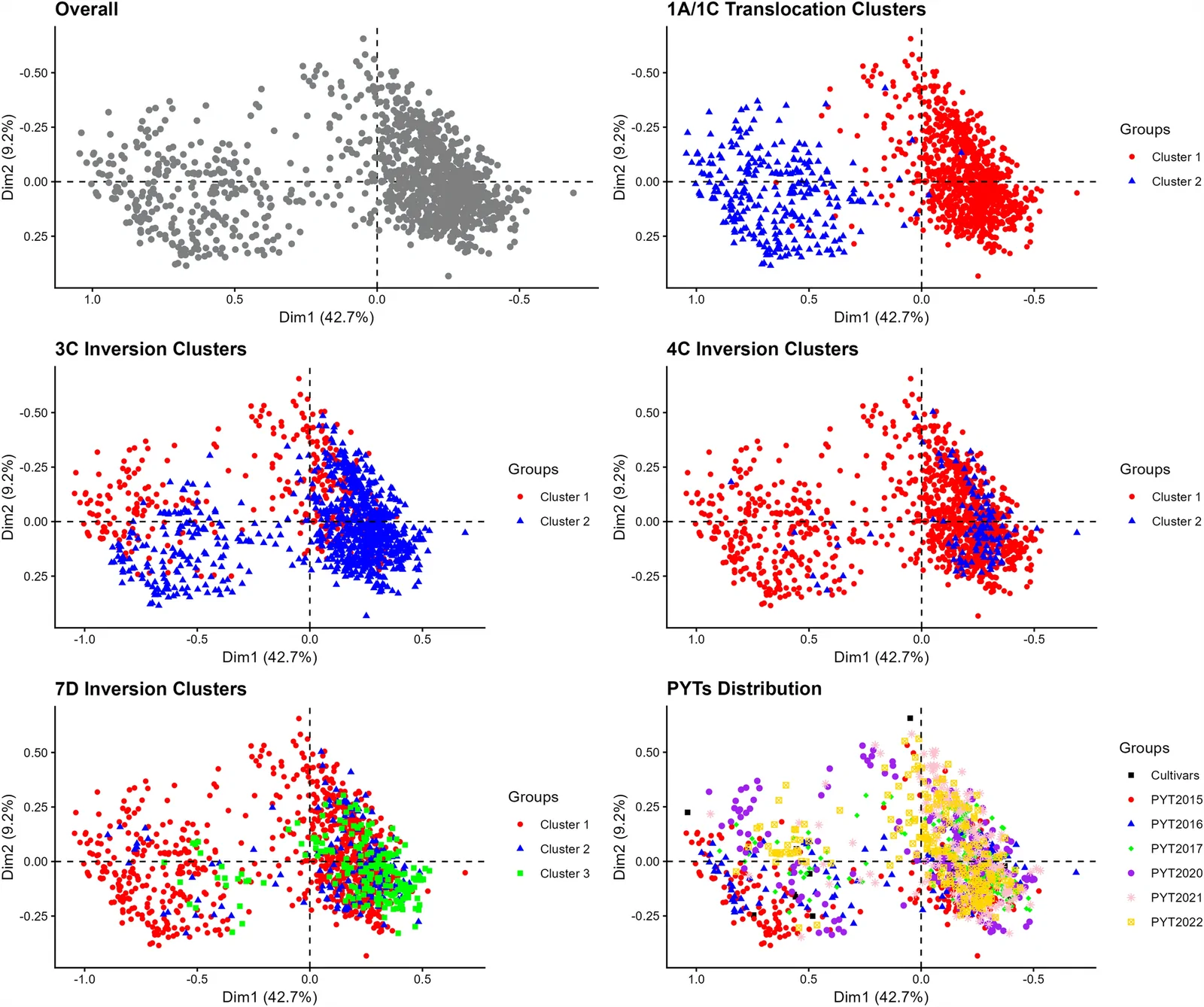
Principal component analysis (PCA) of 1230 advanced oat breeding lines using all genomic markers available (*n* = 9341 SNPs). The PCAs differentiate based on no groupings (Overall), color groupings based on chromosome rearrangement clusters (1A/1C Translocation, 3C Inversion, 4C Inversion, and 7D Inversion), and finally color grouping based on nursery (PYT Year). PYTs = Preliminary Yield Trials

Four major signatures of chromosomal rearrangements (Bekele et al. 2025) in oats were characterized in a set of 1230 breeding lines from the South Dakota State University oat breeding program. The first step in the analysis was to determine the number of haplotypes in those rearrangement regions. For the translocation between chromosome 1A and 1C located at position 420–525 bp on chromosome 1A, the ratio of between-cluster to total sum of squares suggested the presence of two clusters (*K* = 2; 0.942) (Supplementary Table 2), with cluster 2 corresponding to the haplotype found in the synthetic line ‘Amagalon’ (*Avena magna* × *Avena longiglumis*). The first two principal components accounted for 40% of the genetic variance in the PCA conducted within the translocation region (Fig. 1 and Supplementary Table 3). Similarly, for the chromosome inversions located at 80–404 bp on chromosome 3C and at 208–497 on chromosome 4C, two clusters (*K* = 2; 0.911; *K* = 2; 0.964, respectively) were found (Supplementary Table 2), potentially reflecting differentiation between inverted and non-inverted haplotypes. The first two principal components explained 64 and 62% of the genetic variance within the inversion regions on 3C and 4C, respectively (Fig. 1 and Supplementary Table 3). For the inversion on 7D, the ratio of between-cluster to total sum of squares revealed the presence of three clusters (*K* = 3; 0.903) (Supplementary Table 2), with cluster 1 corresponding to the inverted haplotype and cluster 2 and 3 corresponding to two different types of non-inverted haplotypes. The first three principal components accounted for 57% of the variance in the PCAs conducted for the SNPs within the 7D inversion region (Fig. 1 and Supplementary Table 3). These results suggest a moderate to strong local structure in the chromosome rearrangements regions. A strong population structure was observed using the genome-wide set of markers, with the first two principal component dimensions accounting for approximately 52% of the genetic variance (Fig. 2). Additionally, the PCA revealed two large agglomerations of individuals scattered across the plot (Fig. 2). When plotting this PCA based on clusters identified by chromosome rearrangements and nursery origins, the clusters defined by the 1A/1C translocation region were the best indicators of population structure. This is confirmed by most markers located on chromosomes 1A and 1C, which show strong effects on PC1 (Supplementary Fig. 1). Specifically, cluster 1 was predominantly located in the agglomeration on the positive side of dimension 1, while most individuals from cluster 2 were positioned in the agglomeration on the negative side of dimension 1 (Fig. 2). This pattern was not observed for the other chromosome rearrangement clusters, where individuals from some clusters were more frequently spread across both agglomerations (Fig. 2).

Plotting the PCA using the genome-wide set of markers based on the nursery classification revealed a transition, where most of the lines from the most recent PYTs (2021 and 2022) clustered on the negative side of Dimension 1 (Fig. 2), suggesting that line development and selection have been directed toward this agglomeration. This is consistent with the change in frequency of individuals within each cluster type for the 1A/1C translocation across the years (Supplementary Fig. 2 and Supplementary Table 4). In PYT 2015, the clusters were approximately evenly distributed (50:50); however, this balance became increasingly skewed in the later PYTs. In PYT 2021, cluster 1 had the highest frequency, with 200 out of 215 individuals (Supplementary Table 4) classified as cluster 1, representing approximately 93% (Supplementary Fig. 2). In the case of the other chromosomal rearrangement, cluster 2 for the 3C inversion, cluster 1 for the 4C inversion, and cluster 1 for the 7D inversion were consistently predominant across all PYTs (Supplementary Fig. 2). It is worth noting that the proportion of cluster 2 in the 3C inversion was above 60% in all PYTs, while cluster 1 in the 4C inversion exceeded 90%, and cluster 1 in the 7D inversion was consistently above 50% (Supplementary Fig. 2). For the other 7D inversion clusters, the proportion of cluster 3 was higher than that of cluster 2 in most PYTs (Supplementary Fig. 2).

### Linkage disequilibrium analysis on chromosomes with rearrangements

**Fig. 3:**
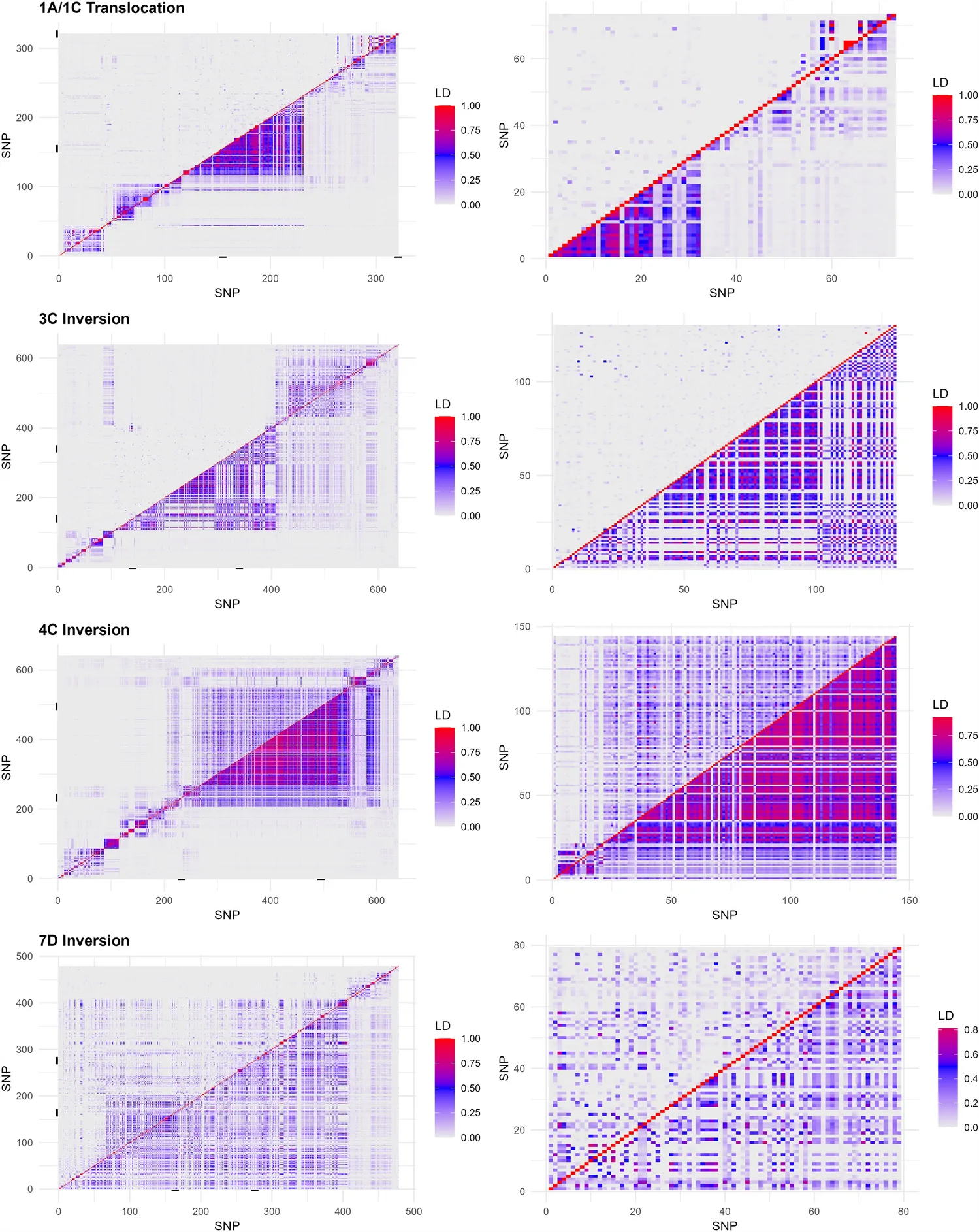
Linkage disequilibrium (LD) in the whole chromosome and in the rearranged region of the respective chromosome. At the top of the LD graphs, individuals from a specific cluster are shown (1A/1C translocation = cluster 2; 3C inversion = cluster 1; 4C = cluster 1; 7D inversion = cluster 3), while at the bottom, the entire set of individuals is displayed. The black squares indicate the boundaries (start and end) of the rearranged regions. The right-side plots show a zoomed-out view of the rearranged regions

**Fig. 4:**
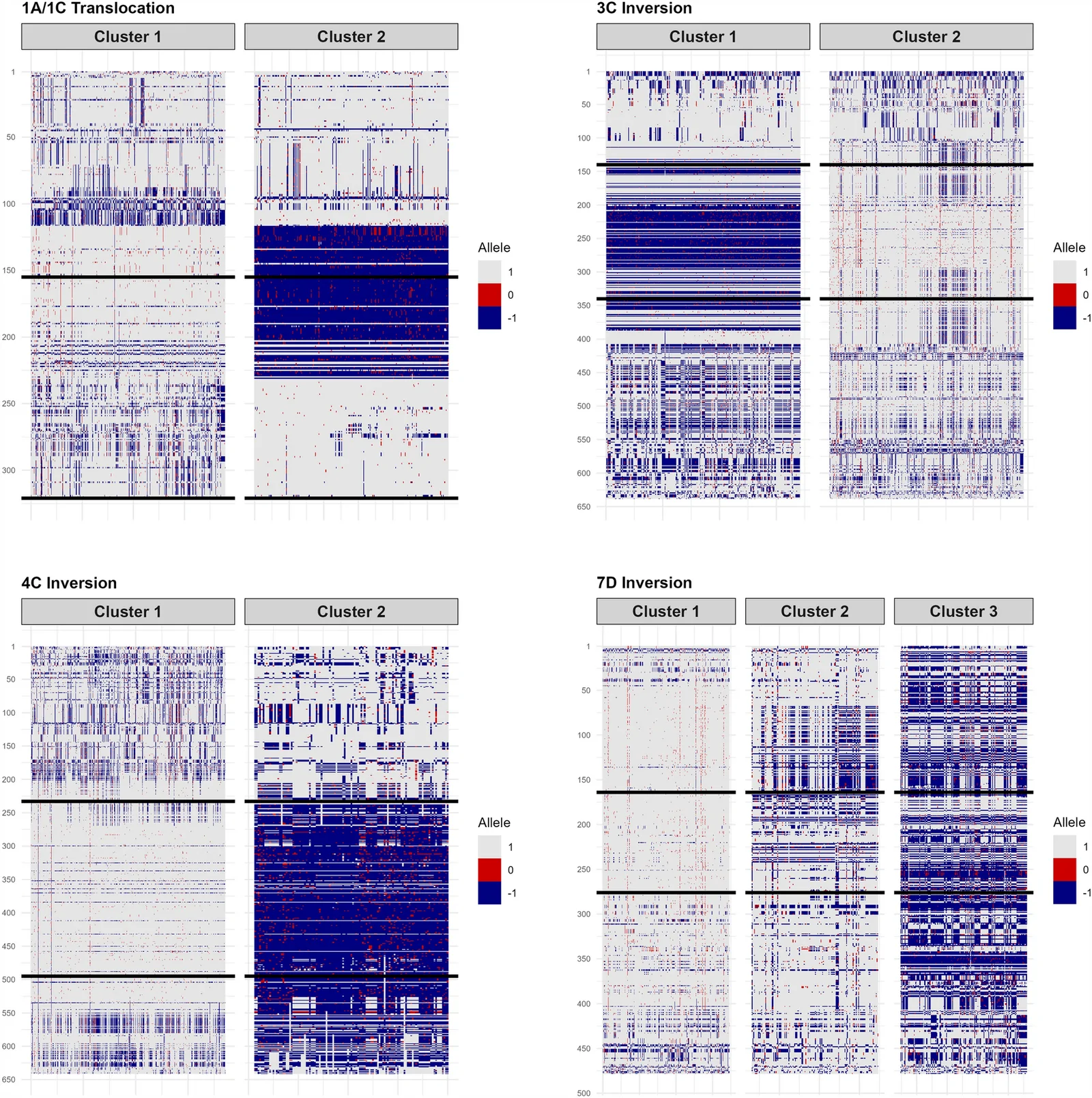
Allele profile of chromosomes containing rearranged regions (top left: Chromosome 1A; top right: Chromosome 3C; bottom left: Chromosome 4C; bottom right: Chromosome 7D). The black lines indicate the boundaries (start and end) of the signatures of the rearranged regions based on Bekele et al. 2025. The *y*-axis shows the SNPs, numbered sequentially according to their order and position on the respective chromosome. Each line on the *x*-axis represents one oat genotype line from its representative cluster, with the corresponding SNP profile coded as 1 = gray, 0 = red, and − 1 = navy

By analyzing linkage disequilibrium (LD) on chromosomes 1A, 3C, 4C, and 7D, which exhibit the chromosomal rearrangements explored in this study, and using the marker data from 1230 advanced breeding lines, we identified specific genomic regions with higher LD values in chromosomes 1A, 3C, and 4C compared to other chromosomal regions. This suggests reduced recombination in these areas (Fig. 3). Interestingly, on chromosome 7D, no specific genomic region exhibited higher LD values compared to other parts of the chromosome; however, the overall LD across the chromosome exceeded 0.1, indicating that even distant markers maintain a considerable degree of linkage (Fig. 3). When running the LD analysis using only the oat breeding lines within the same cluster (i.e., 1A/1C translocation = cluster 2; 3C inversion = cluster 1; 4C = cluster 1; 7D inversion = cluster 3), the previously identified genomic regions with high LD values either disappear or show reduced LD (Fig. 3). As expected, the genomic regions with higher LD values were located in the chromosome rearrangement regions on chromosomes 3C and 4C (Fig. 4), as well as on the rearrangement region of chromosome 7D (Fig. 3). However, only a small portion of the high-LD region overlapped with the chromosome rearrangement region on chromosome 1A (Fig. 3). Using progeny derived from parents assigned to distinct genomic clusters, we conducted a deeper investigation into the genetic diversity of these chromosomes following the LD analysis. Within the chromosomal rearrangement regions, a lack of recombination was observed. The progenies, representing selected lines from the breeding process, essentially retained the same marker profile as one of the parents, with no recombination occurring in these regions, regardless of which chromosomal rearrangement region was analyzed (Supplementary Fig. 3). The lack of recombination is also supported by differences in marker variant composition and distribution among clusters within the same chromosome rearrangements when analyzing the entire dataset of lines (Fig. 4). In addition, the 1A/1C translocation cluster 1, 3C inversion cluster 2, 4C inversion cluster 2, and 7D inversion cluster 3, respectively, exhibited higher genetic variability compared to the other cluster(s) within their respective chromosomal rearrangement regions (Supplementary Fig. 4).

### Phenotypic variability of oat grain yield and adaptation traits across chromosome rearrangement-based clusters

Results from the ANOVA for the entire dataset revealed that the 1A/1C translocation had a significant effect on CRS, GYL, and HDT (Table 1). Overall, cluster 2 (individuals with the 1A/1C translocation) showed higher GYL, higher CRS, higher PHT, and later HDT than individuals from cluster 1 (no translocation) (Supplementary Fig. 5). The overall analysis also revealed a significant interaction between the haplotype for 1A/1C and the PYT for GYL, HDT, and PHT (Table 1). When analyzing each PYT set individually, results agreed on the effect of the 1A/1C translocation on CRS, PHT and HDT; however, it was cluster 1 (no translocation signature) that had significantly higher GYL for PYT2015 and PYT2016 (Supplementary Fig. 6).

**Table 1:** Summary of analysis of variance for crown rust severity, grain yield, heading date, and plant height across oat structural variations and preliminary yield trials (PYTs)

Trait	Source	*df*	SS	MS	*F* value	*p*-value
Crown rust severity	1A/1C Translocation (1A/1C)	1	5450	5450	36.642	2.07E−09***
	3C Inversion (3C)	1	132	132	0.887	0.346
	4C Inversion (4C)	1	228	228	1.535	0.216
	7D inversion (7D)	2	2313	1156.5	7.774	4.49E−04***
	PYT	4	33,263	8315.7	55.910	<2.2e−16***
	1A/1C:PYT	4	795	198	1.336	0.255
	3C:PYT	4	1363	340.7	2.291	0.058
	4C:PYT	4	69	17.3	0.116	0.976
	7D:PYT	8	3274	409.3	2.752	0.005**
Grain yield	1A/1C Translocation (1A/1C)	1	4764947	4764947	16.393	5.54E−05***
	3C Inversion (3C)	1	83382	83382	0.287	0.592
	4C Inversion (4C)	1	932007	932007	3.206	0.074
	7D inversion (7D)	2	3284995	1642498	5.651	0.004**
	PYT	5	193239900	38647980	132.958	<2.2E−16***
	1A/1C:PYT	5	9615252	1923050	6.616	4.54E−06***
	3C:PYT	5	2174646	434929	1.496	0.188
	4C:PYT	5	2804759	560952	1.930	0.087
	7D:PYT	10	11712648	1171265	4.029	1.89E−05***
Heading date	1A/1C Translocation (1A/1C)	1	19.77	19.77	21.241	4.49E−06***
	3C Inversion (3C)	1	10.91	10.91	11.723	6.38E−04***
	4C Inversion (4C)	1	0.55	0.55	0.589	0.443044
	7D inversion (7D)	2	13.81	6.91	7.419	6.29E−04***
	PYT	5	1952	390.4	419.372	<2.2E−16***
	1A/1C:PYT	5	12	2.4	2.578	0.026*
	3C:PYT	5	7.19	1.44	1.545	0.173
	4C:PYT	5	8.32	1.67	1.792	0.112
	7D:PYT	10	36.4	3.64	3.910	3.21E−05***
Plant height	1A/1C Translocation (1A/1C)	1	326.1	326.1	18.795	1.57E−05***
	3C Inversion (3C)	1	1304.7	1304.68	75.206	<2.2E−16***
	4C Inversion (4C)	1	197.3	197.3	11.373	7.69E−04***
	7D inversion (7D)	2	595.8	279.9	17.171	4.45E−08***
	PYT	5	13,164.3	2632.86	151.766	<2.2E−16***
	1A/1C:PYT	5	390.8	78.16	4.505	4.74E−04***
	3C:PYT	5	448.4	89.68	5.169	1.07E−04***
	4C:PYT	5	109.9	21.99	1.267	0.276
	7D:PYT	10	262.0	26.20	1.510	0.130

*df* Degrees of freedom; *SS* sum of squares; *MS* mean squares. ***indicates a *p*-value less than 0.001, ** indicates a p-value less than 0.01, * indicates a *p*-value less than 0.05

The 3C inversion had a significant effect on HDT and PHT, with a significant PYT × haplotype interaction when data were analyzed across PYTs (Table 1). Cluster 2 of the 3C inversion had significantly earlier HDT and shorter PHT than cluster 1 (Supplementary Fig. 5). This was also observed for individual PYT analyses (Supplementary Fig. 7).

In the overall analysis, the 4C inversion affected only PHT and had no effect on any other traits (Table 1 and Supplementary Fig. 5). This was confirmed in the individual PYT analyses, with cluster 2 being taller than cluster 1 in PYT 2020 and PYT 2021 (Supplementary Fig. 8). While the inversion on chromosome 4C did not have any effect on CRS with either analysis, a significant effect on GYL (PYT 2021) and HDT (PYT2016 and PYT 2020) was detected for some individual PYT analyses.

The 7D inversion had a significant effect on all traits and these effects were detected in both analyses. There were also significant interactions between haplotype and PYT for all traits (Table 1). The overall analysis revealed that cluster 3 had lower CRS than clusters 1 and 2, higher GYL than cluster 2, and earlier maturity than cluster1, while cluster 1 was taller than the other two clusters (Supplementary Fig. 5). We also conducted an analysis for each PYT separately. The results were consistent between the two types of analyses for CRS, HDT, and PHT; however, the effect of the 7D insertion on GYL was inconsistent (Supplementary Fig. 9). Cluster 3 produced higher GYL than cluster 2 for PYT2015 and 2016, and than cluster 1 for PYT 2015 and PYT 2020, but lower GYL than cluster 1 in PYT2017.

### Genome-wide association studies of oat grain yield and adaptation traits based on chromosomal rearrangement clusters

**Fig. 5:**
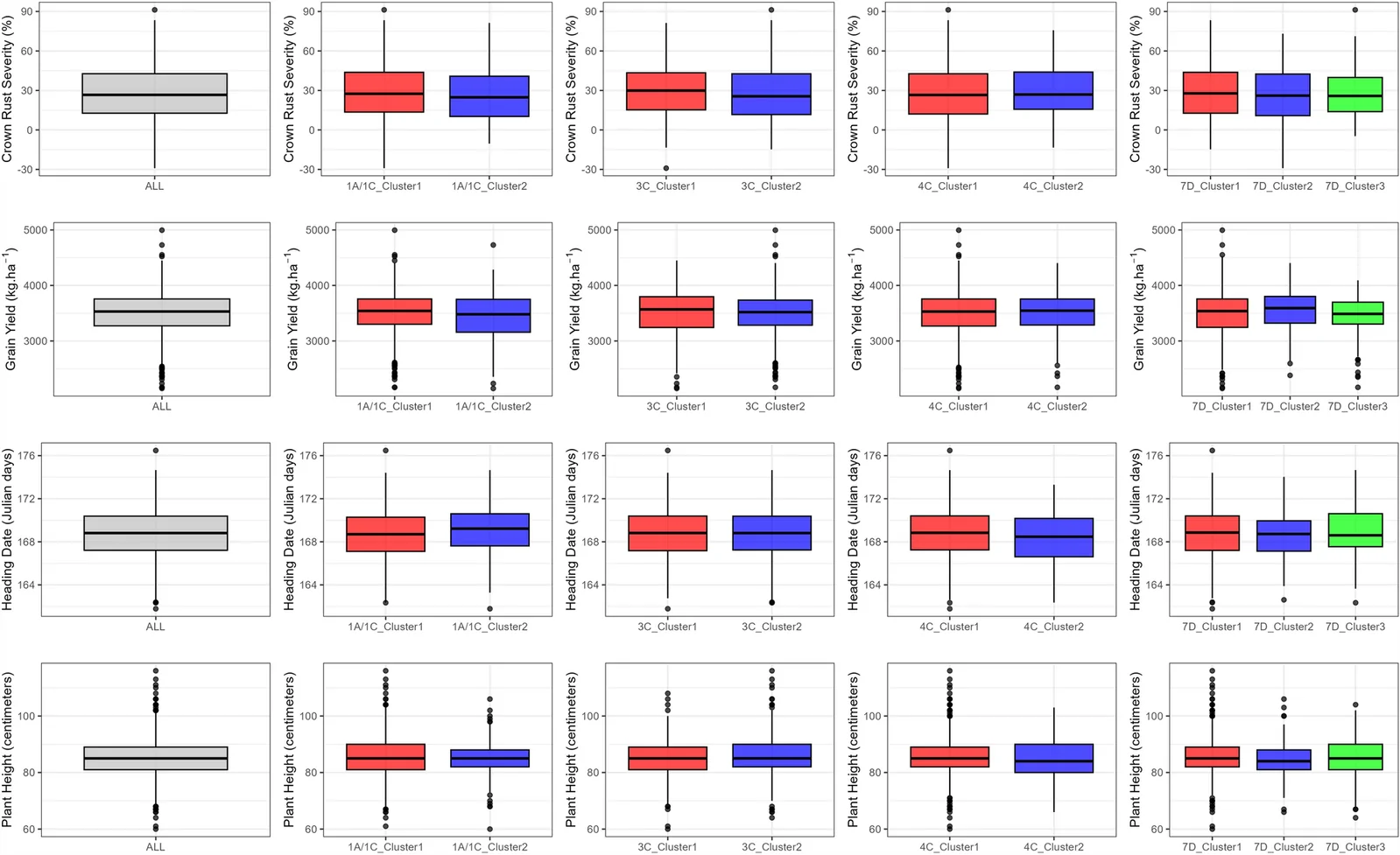
Phenotypic distribution across the entire population and within chromosomal rearrangement clusters of crown rust severity, grain yield, heading date, and plant height using across environment BLUEs (Eq. 2)

**Fig. 6:**
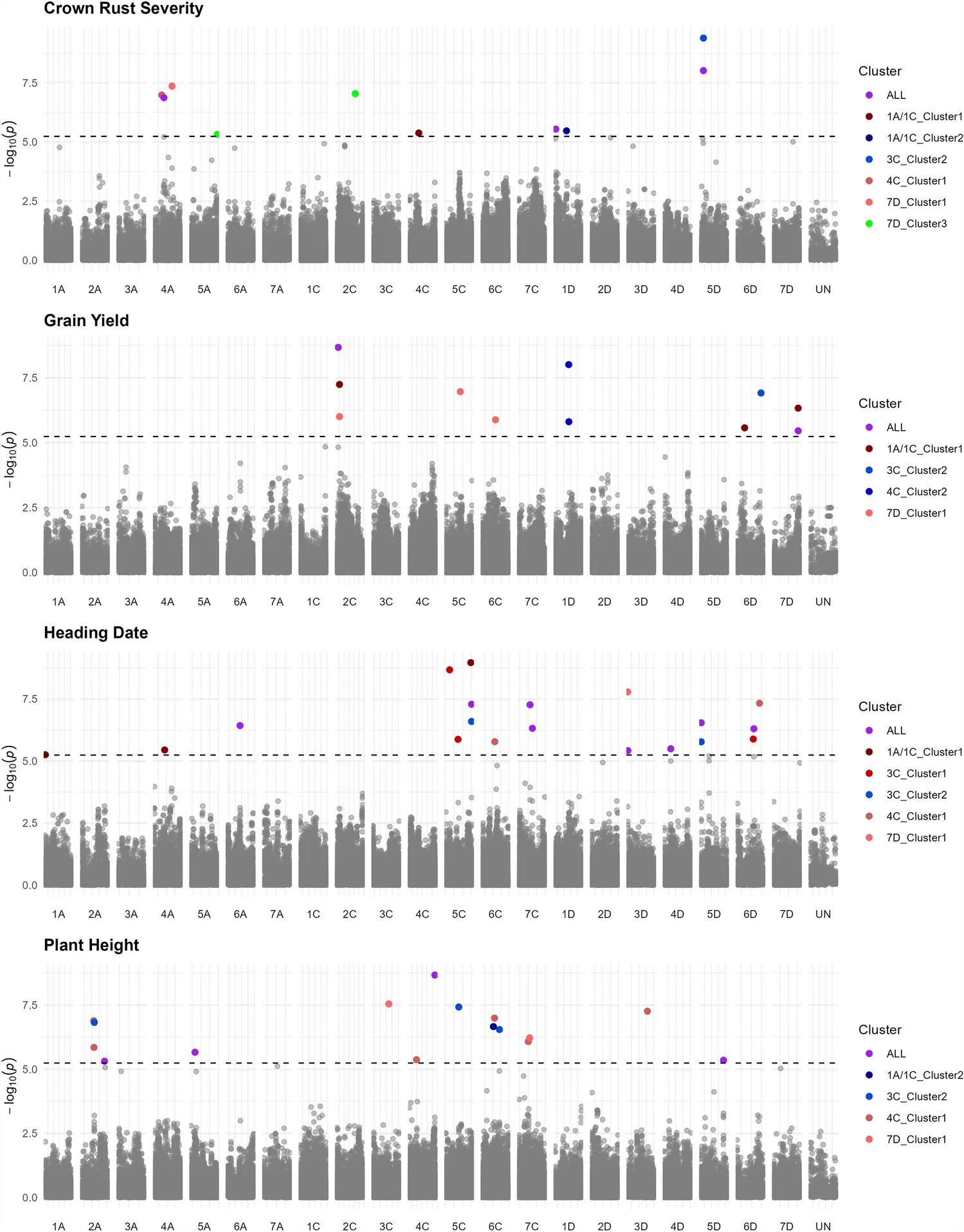
Manhattan plots showing significant SNPs markers associated with crown rust severity, grain yield, heading date, and plant height. The *x*-axis represents the oat chromosomes, while the *y*-axis shows the statistical significance $\left(-{\mathrm{log}}_{10}(p)\right)$ of marker–trait associations. Each dot represents a SNP marker; gray dots indicate nonsignificant associations, whereas colored dots indicate significant associations. Different colors represent the subpopulation cluster in which the association was identified. ALL = full dataset (*n* = 1230)

To identify the effect of those chromosome rearrangements on GWAS results, we followed two strategies. First, we conducted GWAS including chromosomal rearrangements as covariates using the full dataset (*n* = 1230). This approach did not identify results that differed from those obtained without covariates (Supplementary Table 5). Second, we conducted GWAS within the subpopulations of each SV cluster and compared those results with the entire population GWAS. Boxplots for all four traits were developed to assess data distribution within each subpopulation (Fig. 5). Although some clusters contain a small number of individuals (Supplementary Table 4), their distribution and phenotypic range remained normally distributed with similar range in values to those observed in the entire set of individuals (*n* = 1230) (Fig. 5). Overall, 40 GWASs were conducted, leading to the detection of 47 SNP markers significantly associated with the four traits analyzed (Table 2 and Fig. 6). Some associations were identified on chromosomes harboring chromosomes rearrangements, in some cases near those rearranged regions, however, no associations were found inside the regions of chromosome rearrangements (Table 2), whether the GWAS was performed across the entire genotype set or within cluster groups corresponding to a specific chromosome rearrangement haplotype (Table 2). On chromosome 1A, using only the individuals classified in cluster 1 for the 1A/1C translocation (*n* = 952), the ‘closest’ marker found to be associated with one of the four traits (HDT) was located at 10.95 Mbp (positioned 410 Mbp distal to one boundary of the 1A/1C translocation) (Table 2 and Fig. 6). A marker located at 448.55 Mbp on chromosome 3C, approximately 44 Mbp away from one border of the 3C inversion region (404 Mbp), was associated with PHT (Table 2 and Fig. 6). However, this association was observed only in the subpopulation of individuals within the 7D cluster 1 (*n* = 865) (Table 2). On chromosome 4C, two markers were associated with PHT (Table 2 and Fig. 7). One of the markers was identified using the 4C inversion cluster 1 (*n* = 1113) (PHT; 139.81 Mbp), while another one was identified using the whole population (*n* = 1230) and is located at 639 Mbp, approximately 142 Mbp away from one of the borders of the 4C inversion region (Table 2). On chromosome 7D, two markers were identified. One of them, located at 475 Mbp, was detected twice: once using the whole population and once using the 1A/1C translocation cluster 1 (*n* = 952). The other marker, located at 481.33 Mbp, was identified through the 4C cluster 2 subpopulation (*n* = 117). Both markers on chromosome 7D were associated with GYL.

**Table 2:** Genomic regions controlling oat grain yield (GYL), crown rust severity (CRS), heading date (HDT), and plant height (PHT) identified in different clusters of advanced breeding lines

Marker	Chromosome	Position (Mbp)	MAF (%)	Cluster	Trait	-log *p* value
Schr1A_9291409	1A	10.95	0.18	1A-Cl1	HDT	5.25
Schr2A_394650928	2A	388.56	0.25	3C-Cl2	PHT	6.82
Schr2A_394650928	2A	388.56	0.32	4C-Cl1	PHT	6.89
Schr7D_445472321	2A	446.55	0.22	ALL	PHT	5.31
Schr7D_445472321	2A	446.55	0.22	4C-Cl1	PHT	5.85
Schr4A_283178147	4A	287.83	0.22	ALL	CRS	6.88
Schr4A_283178147	4A	287.83	0.21	4C-Cl1	CRS	6.99
Schr4A_285021567	4A	289.67	0.15	1A-Cl1	HDT	5.45
Schr4A_392463679	4A	393.29	0.37	7D-Cl1	CRS	7.37
Schr5A_100781446	5A	103.54	0.12	ALL	PHT	5.66
Schr5A_482906352	5A	474.89	0.25	7D-Cl3	CRS	5.33
Schr6A_307431632	6A	305.36	0.41	ALL	HDT	6.43
Schr2C_19640030	2C	19.38	0.17	ALL	GYL	8.67
Schr2C_50424356	2C	47.04	0.07	1A-Cl1	GYL	7.25
Schr2C_50424356	2C	47.04	0.10	7D-Cl1	GYL	6.01
Schr2C_498929506	2C	461.86	0.48	7D-Cl3	CRS	7.05
Schr3C_507228869	3C	448.55	0.06	7D-Cl1	PHT	7.55
Schr4C_135770760	4C	139.81	0.08	4C-Cl1	PHT	5.37
Schr4C_188000042	4C	188.18	0.04	1A-Cl1	CRS	5.38
Schr4C_683988410	4C	639.12	0.18	ALL	PHT	8.67
Schr5C_29176731	5C	29.37	0.21	3C-Cl1	HDT	8.68
Schr5C_399337024	5C	377.48	0.18	3C-Cl1	HDT	5.87
Schr5C_457933753	5C	427.38	0.41	3C-Cl2	PHT	7.42
Schr5C_503660327	5C	471.19	0.24	7D-Cl1	GYL	6.97
Schr5C_602906451	5C	562.63	0.08	1A-Cl1	HDT	8.97
Schr5C_600779048	5C	565.13	0.06	ALL	HDT	7.29
Schr5C_600779048	5C	565.13	0.06	3C-Cl2	HDT	6.59
Schr6C_52466224	6C	49.97	0.12	4C-Cl1	HDT	5.78
Schr6C_89856517	6C	84.02	0.05	1A-Cl2	PHT	6.66
Schr6C_101936730	6C	96.00	0.14	7D-Cl1	GYL	5.89
Schr6C_108136977	6C	101.89	0.12	4C-Cl1	PHT	6.99
Schr6C_127356397	6C	120.16	0.06	3C-Cl2	PHT	6.55
Schr7C_90862778	7C	83.04	0.30	7D-Cl1	PHT	6.22
Schr7C_95267361	7C	87.31	0.23	ALL	HDT	7.27
Schr7C_148450207	7C	136.59	0.18	ALL	HDT	6.32
Schr7C_549921822	7C	501.05	0.17	4C-Cl1	PHT	6.08
Schr1D_15684404	1D	17.56	0.18	ALL	CRS	5.55
Schr1D_336412425	1D	327.31	0.26	1A-Cl2	CRS	5.47
Schr1D_449068074	1D	435.54	0.08	4C-Cl2	GYL	8.01
Schr3D_8883976	3D	0.26	0.21	7D-Cl1	HDT	7.78
Schr3D_8646835	3D	0.50	0.19	ALL	HDT	5.42
Schr3D_451241889	3D	427.55	0.39	4C-Cl1	PHT	7.26
Schr4D_344166510	4D	307.55	0.31	ALL	HDT	5.49
Schr5D_15057253	5D	17.25	0.12	ALL	HDT	6.54
Schr5D_15057253	5D	17.25	0.13	3C-Cl2	HDT	5.77
Schr5D_42971356	5D	45.34	0.39	ALL	CRS	8.02
Schr5D_42971359	5D	45.34	0.39	3C-Cl2	CRS	9.40
Schr5D_483773546	5D	474.58	0.08	ALL	PHT	5.35
Schr6D_223894830	6D	216.49	0.07	1A-Cl1	GYL	5.57
Schr6D_276198585	6D	267.71	0.30	3C-Cl1	HDT	5.89
Schr6D_276198599	6D	267.71	0.16	ALL	HDT	6.30
Schr6D_276198599	6D	267.71	0.16	4C-Cl1	HDT	7.33
Schr6D_289669647	6D	280.29	0.13	3C-Cl2	GYL	6.92
Schr7D_513559679	7D	475.17	0.19	ALL	GYL	5.46
Schr7D_513559679	7D	475.17	0.22	1A-Cl1	GYL	6.33
Schr7D_520360548	7D	481.33	0.08	4C-Cl2	GYL	5.81

*ALL* full dataset (*n* = 1230); *Cl* Cluster; *Mbp* mega-base pair

Most associations identified were unique for the whole population (e.g., ‘Schr4D_344166510’ associated with HDT in the chromosome 4D) or were in close genomic proximity to those identified using cluster-based populations. However, some associations and genomic regions were uniquely identified through the cluster-based subpopulations (Table 2). For instance, all the associations identified on chromosome 6C were detected only through the cluster-based subpopulations (Table 2). Almost all associations identified in this study have been previously reported in the literature and are located near important adaptation genes (Supplementary Table 6).

### Genomic predictions within chromosomal rearrangement clusters

Genomic prediction strategies using cross-validation schemes were developed to assess whether higher prediction accuracy for the analyzed traits could be achieved by accounting for chromosome rearrangement haplotypes. Two strategies were evaluated. First, genomic prediction models were developed using the chromosome rearrangement haplotype as a covariate. This strategy did not improve the prediction accuracies in comparison with those obtained using the whole set of lines without accounting for chromosomal rearrangements (Supplementary Table 7). Second, prediction models were developed specifically for each subpopulation based on chromosomal rearrangements. Using this strategy, higher prediction accuracy was obtained in some cases. For GYL, an accuracy of 0.31 was obtained in the 1A/1C Cluster 2 (vs. *r* = 0.20 for the whole population); for CRS, an accuracy of 0.45 was obtained in the 7D Cluster 3 (vs. *r* = 030 for the whole population); for HDT, an accuracy of 0.34 was obtained in the 4C Cluster 2 (vs. *r* = 0.27 for the whole population); and for PHT, an accuracy of 0.35 was obtained in the 7D Cluster 2 (vs. *r* = 0.26 for the whole population) (Table 3). In most cases, the prediction accuracy obtained for models developed within clusters was close to the prediction accuracy obtained for models developed for the entire population. In a few cases, models developed for specific clusters had low prediction accuracy in comparison with models developed based on the entire population (i.e., 3C cluster 1 for GYL and PHT; 7D cluster 3 for HDT and PHT).

**Table 3:** Predictive accuracies for genomic prediction models using cluster-based populations for crown rust severity (CRS), grain yield (GYL), heading date (HDT), and plant height (PHT)

Cluster	*n*	GYL	CRS	HDT	PHT
		Accuracy (*r*)			
ALL	1230	0.20	0.30	0.27	0.26
1A/1C Cluster 1	949	0.19	0.32	0.24	0.28
1A/1C Cluster 2	281	**0.31**	0.33	0.27	0.12
3C Cluster 1	342	0.12	0.25	0.30	0.06
3C Cluster 2	888	0.22	0.28	0.24	0.25
4C Cluster 1	1113	0.20	0.29	0.25	0.25
4C Cluster 2	117	0.23	0.32	**0.34**	0.25
7D Cluster 1	865	0.22	0.33	0.31	0.27
7D Cluster 2	158	0.17	0.20	0.26	**0.35**
7D Cluster 3	207	0.17	**0.45**	0.15	0.18

*n* Number of individuals

Bold indicates the highest accuracy value for each trait

## Discussion

In *Avena* species, chromosomal rearrangements are a common phenomenon in their evolutionary history and are considered one of the main drivers of speciation, altered gene count per genome, and changes in gene expression (Kamal et al. 2022; Tinker et al. 2022; Tomaszewska et al. 2022). Genomic signatures of these structural variants are still observed in advanced breeding lines (Avni et al. 2025; Bekele et al. 2025), serving as one of the sources of genetic variability among breeding programs. This was confirmed in the SDSU advanced oat breeding germplasm by the PCA analysis conducted in this study. The PCA evaluation of four genomic regions potentially harboring chromosomal rearrangements revealed distinct clusters, which together explained more than 40% of the genetic variance (Fig. 1 and Supplementary Fig. 1). Lines from different clusters have markedly different genetic profiles in those specific regions (Fig. 4). Genetic diversity is essential for any plant breeding program, as selection is only effective in a group of individuals that differ genetically (Swarup et al. 2021). However, thus far, it has been unclear how the genetic differences within the chromosomal rearrangement regions have been exploited in oat breeding programs.

Among the chromosome’s rearrangements evaluated, the 1A/1C translocation was the most prominent, with its presence/absence overlapping well with the overall genetic landscape present among our set of breeding lines (Fig. 2). The loadings (eigenvector coefficients) for markers on PC1 (42.7% of the variance explained) are strongly associated with markers on chromosomes 1A and 1C (Supplementary Fig. 1). This suggests that the 1A/1C plays a large role in the population structure in our breeding population. Additionally, one of the two haplotypes for this structural variant appears to have been subject to selection within the last 10 years of breeding activities in the program, as indicated by a clear decline in the presence of cluster 2 associated with this rearrangement in the most recent SDSU PYTs (Fig. 2 and Supplementary Fig. 2). Cluster 2 is associated with Amagalon-derived lines, whose genetics were introgressed in the SDSU oat breeding germplasm through crosses with breeding lines from North Dakota State University oat breeding program, including cultivar HiFi (McMullen et al. 2005). One of the main reasons behind these crosses was to exploit gene Pc91, which previously conferred resistance to crown rust and originated from *Avena magna* CI 8330, one of Amagalon’s parents (Park et al. 2022). Our phenotypic data indicate that cluster 2 (Amagalon-type lines) was more susceptible to crown rust than cluster 1 (Supplementary Figs. 5 and 6). This is not unexpected as Pc91 was no longer effective against crown rust races prevalent in South Dakota when our data for this study were collected. This could partially explain why no associations for CRS were identified in the 1A/1C translocation region (Table 2), where Pc91 is located (McCartney et al. 2011; Gnanesh et al. 2014; Esvelt Klos et al. 2017; Maughan et al. 2019). The limited recombination frequency in the translocation region combined with the use of crown rust resistance from other sources which did not carry the translocation and a strong selection pressure for crown rust resistance in the breeding program could have been reasons for the decrease in the frequency of breeding lines belonging to cluster 2 in the most recent PYTs. This scenario could be beneficial if the Amagalon type does not carry other beneficial alleles. Phenotypic data for traits other than CRS demonstrated that the Amagalon types generally showed inferior agronomic performance. However, they still presented superior GYL in PYT 2015 and PYT 2016 (Supplementary Figs. 5 and 6), and the presence of beneficial alleles for traits not analyzed in this study cannot be ruled out. Nevertheless, the loss of this genetic variability in the program could limit genetic gain if other beneficial alleles were located at gene loci on the translocated portion of the chromosome. Our results demonstrate an interesting case of breeding germplasm genetic stratification due to introgression of wild genetics in oats. Comparable patterns have also been documented in other crops, including cassava (*Manihot esculenta Crantz*) (Wolfe et al. 2019) and wheat (*Triticum aestivum L.*) (Keilwagen et al. 2022).

The clusters in the rearranged regions of the other chromosomes show a predominant cluster, with no indication of changes in their proportions over time (Supplementary Fig. 2). The inversions on chromosomes 3C and 4C are not fully characterized (Avni et al. 2025; Bekele et al. 2025). Our results show high genetic variability between clusters not only within the regions affected by these inversions but also in other parts of these chromosomes (Fig. 4). Interestingly, for chromosome 3C, few associations have been reported in the literature (Wight et al. 2024), and these are typically located at the extreme ends of the chromosome rather than in the pericentromeric region where the inversion signature is located. Examples include QTLs for winter hardiness (Tumino et al. 2016) and β-glucan content (Asoro et al. 2013), both positioned around 1 Mbp, as well as QTLs for crown rust resistance located between 582 and 632 Mbp (McNish et al. 2020). It is important to mention that most of them were matched to the OT3098 v2 physical map, which may cause slight variation when compared to the Sang v1 physical map. In this study, a marker relatively close to the 3C chromosome inversion region, when compared to associations previously reported in the literature, was identified for PHT (Table 2). However, this association was not found in any of the 3C cluster subpopulations, but rather in the 7D inversion cluster 1 (Table 2), representing a case where the association was likely 'masked' when analyzing the whole population. The phenotypic data suggest a potentially strong association, as the two clusters for 3C are statistically differentiated for PHT in the overall analysis, as well as in the individual PYT analyses, with the exception of PYT 2016 (Supplementary Figs. 5 and 7). Interestingly, the phenotypic data also suggest some relationship with HDT (Supplementary Fig. 5 and Table 1); however, no association was observed for this trait on chromosome 3C (Fig. 6). Similarly to the 3C inversion scenario, associations reported in the literature are rarely found within the 4C inversion genomic regions (Wight et al. 2024), where QTLs identified in earlier genomic studies, based on linkage groups, span large regions. The two markers identified in this study on chromosome 4C, associated with PHT, are not located in or near the inverted regions (Table 2 and Fig. 6), despite phenotypic data suggesting a strong relationship between this structural variation and the traits (Supplementary Fig. 5 and Table 1).

The rearrangement region on chromosome 7D was the only one in our study classified as having more than two clusters (*K* = 3; silhouette score = 0.903) (Fig. 1, Supplementary Table 2), suggesting greater genetic diversity associated with this inversion signature compared to the other structural variants analyzed. This was confirmed by examining the crosses and progenies between clusters: Cluster 1 primarily had markers in the inverted region coded as 1, cluster 3 had most markers coded as −1, while cluster 2 showed a mix of markers coded as 1 and −1 (Fig. 4), suggesting that recombination events between the inverted and non-inverted haplotypes likely occurred at some point. Chromosome 7D has historically been associated with low recombination (Tinker et al. 2022) and may harbor important adaptation genes, primarily related to heading date, such as *VRN*3 and *CO*1 (Tinker et al. 2022), as well as other key loci linked to biotic stress resistance and milling quality (Wight et al. 2024; Bazzer et al. 2025). It aligns with our phenotypic data, as the 7D inversion was highly significantly associated with the four traits analyzed in this study, both in the overall and individual PYT analyses (Supplementary Figs. 5 and 9, and Table 1), confirming the importance of this chromosome and suggesting that this inversion may have a potential impact on key oat traits. However, consistent with the literature, no associations were identified within the 7D chromosome inversion region in this study. The closest association was for GYL, found using the whole population and the 1A/1C cluster 1 individuals, located approximately 63 Mbp away from the inversion signature region (Table 2 and Supplementary Table 1). In contrast to observations for the other chromosome rearrangements regions, where high LD is observed mainly in the chromosome rearrangement region, the chromosome 7D in our dataset presents moderate to high LD values across the whole chromosome (Fig. 3), meaning that distant markers in the chromosomes are linked. These results indicate that chromosome 7D is not only a key genetic source but also a highly complex chromosome in oats, where the signature inversion region is completely different from the other chromosomes, and the identification of genes can be drastically affected by LD, at least in the SDSU germplasm.

As previously mentioned, except for chromosome 7D, all chromosomes with genomic signatures of rearrangements show a specific region with high LD compared to other parts of the chromosome (Fig. 3). While such patterns can arise from reduced recombination within rearranged regions, they can also be generated by population structure alone. When alleles differ in frequency across rearrangement-based clusters, markers can appear strongly associated even if unlinked. For the inversions on chromosomes 3C and 4C, the regions of high LD align precisely with the structural variant signatures. However, for the 1A/1C translocation, only part of the structural variant delineated based on Bekele et al. (2025) overlaps with the high-LD region (Fig. 3), suggesting that the translocation signature may be underestimated in this germplasm. Further research is needed to understand the underlying reasons. As expected, these genomic regions with high LD show considerable decreases in LD values when analyzed within clusters (Fig. 3). These results confirm that the rearrangements are potentially generating population structure and recombination is more likely to occur between individuals with similar chromosomal rearrangements (i.e., within the same cluster) than between those from different clusters. In relation to the low recombination, this was further supported by observations of progenies derived from crosses performed between individuals belonging to different clusters, where no recombination was detected in the rearranged regions (Supplementary Fig. 3). This suggests that recombinants are rare. It may therefore be ideal to prioritize crosses among individuals from the same cluster to target recombination in those regions, especially in chromosome 7D, where important genes are located. For this approach to be effective, parents belonging to the same cluster need to exhibit some allelic variability across the inversion regions such as was observed, for example, for the cluster 3 (Supplementary Fig. 3). Nevertheless, restricting crosses to individuals within the same cluster would limit the opportunity to combine the best alleles from different clusters. Thus, another possibility is the potential exploitation of targeted recombination (Ru and Bernardo 2019) through biotechnological methods, particularly in crosses between different clusters, aiming to develop recombinants in specific genomic regions. However, this approach remains largely theoretical rather than a routine practice in plant breeding. Additionally, our results suggest that these chromosomal rearrangement regions may not harbor important loci (Table 2), imply that targeted recombination in these areas could result in neutral effects or low genetic gains. However, such recombination could break repulsion phase linkages within chromosomal rearrangement regions, thereby exposing hidden causal variation. This, in turn, could improve the detection of associations with major traits and lead to more precise gene localization within those chromosomal regions.

Overall, despite the phenotypic data analyses indicating that those rearrangements had a significant effect on the four agronomic traits considered in this study, GWAS analyses did not detect any significant association between the genotype and the phenotype within the four regions of chromosome rearrangements evaluated in this study. The atypical recombination patterns in the rearrangement regions likely hindered our ability to detect significant associations when the analysis was conducted on the full population. Although recombination may still occur within subpopulations, the genetic variability is reduced to the degree existing within each haplotype, also limiting the ability to detect association between genotype and phenotype. If a gene responsible for the phenotypic variation is present in only one haplotype associated with the rearrangement, the GWAS signal might not be detected within the chromosome rearrangement region but may be observed near the extremity of the rearrangement region when analyzing the full population because of the lack of recombination within the region. If the genetic variability existing within one haplotype remains sufficient, one could expect to detect a significant association within that region in closer proximity of the gene. However, our GWAS results did not detect significant association either within the region or in proximity to the region. Several factors may have influenced the association between phenotype and genotype, ranging from the actual small effect or absence of key loci within these rearranged regions to the genetic complexity of the chromosomes involved, particularly chromosome 7D. Nevertheless, our GWAS results confirmed important regions previously associated with adaptation traits and showed that the breeding populations used in this study, with their genetic diversity, are valuable for association studies. For instance, our results align with the potential location of *VRN*1 on chromosome 4A (with markers associated with CRS and HDT in the 287–289 Mbp range) and 4D (with markers associated with HDT located at 307 Mbp) as reported by Tinker et al. (2022) (Supplementary Table 5). Additionally, an important genomic region on chromosome 6D, previously associated with heading date and potentially harboring the genes *CslF*9/11, related to β-glucan (Tinker et al. 2022), was also identified in our study, through an association with GYL and HDT, with markers ranging from 217 to 280 Mbp (Table 2 and Supplementary Table 5), reinforcing the importance of this genomic region and highlighting the value of understanding subpopulation genetics in a broader context for association studies (Table 2 and Supplementary Table 5).

Optimizing training populations for specific testing populations has been one of the main research areas targeting improved genomic predictions (Asoro et al. 2011; Berro et al. 2019; Sørensen et al. 2023). Population structure arising from distinct subpopulations can negatively affect genomic predictions, as high number of differences in allele frequencies across the genome may lead to spurious associations between genetic markers and phenotypic variation (Isidro et al. 2015). Therefore, developing genomic prediction models for the subpopulations based on the chromosome’s arrangements instead of the whole population may help avoid confounding factors and achieve higher accuracies. The genomic prediction models based on the 1A/1C cluster translocation received extra attention, as the overall PC1 effect is due to markers from the 1A and 1C chromosomes (Supplementary Fig. 2). Interestingly, for GYL, predictions within the 1A/1C cluster 2 achieved the highest accuracy (*r* = 0.31) among all models, particularly when compared to 1A/1C cluster 1 (*r* = 0.19) and the whole set of lines (*r* = 0.20) (Table 3). From a practical standpoint, this could represent an important strategy for the SDSU oat breeding program. Cluster 2 carries Amagalon genetics and was inferior for CRS and HDT. Therefore, having a model that can more accurately identify superior lines within this subpopulation could help unlock genetic potential and improve selection within this group. This same idea may be applied to other clusters and traits to a certain extent, as the cluster-based cross-validation scheme achieved the highest prediction accuracy. However, the other respective clusters showed very low accuracies. For example, in the case of CRS, the prediction accuracy was high within 7D cluster 3 (*r* = 0.45), but much lower in cluster 2 (*r* = 0.20), compared to the accuracy obtained using cross-validation across all lines (*r* = 0.30) (Table 3). It is worth mentioning that within-cluster genomic predictions could be low due to population size, as smaller populations are expected, under certain conditions, to have lower accuracies. Additionally, some limitations must be considered, such as the complexity of managing multiple prediction models in a practical breeding program and the potential decline in accuracy over time if the proportion of certain clusters decreases. Nevertheless, these results demonstrate the potential negative effects of chromosomal rearrangement signatures in a prediction pipeline due to the formation of subpopulations.

## Conclusion

In summary, population structure and linkage disequilibrium analyses using an oat breeding dataset of 1230 lines revealed genetic variability and low recombination in regions associated with chromosomal rearrangements. These findings contribute to a deeper understanding of oat genomics and how it has been directly and indirectly leveraged in an oat breeding program. Although GWAS did not identify associations within the rearranged regions, we observed slight differences in associated genomic regions when comparing the whole population to cluster-based populations, as well as when comparing among cluster-based populations. This underscores the importance of understanding the sources of genetic variability among individuals and their potential impact on association mapping. Genomic predictions within cluster-based groups of individuals outperformed predictions based on the entire population, indicating that subpopulations, due to chromosomal rearrangements in this study, can act as barriers to achieving higher prediction accuracy for multiple traits in oats.

## Supplementary Information

Below is the link to the electronic supplementary material.

Supplementary file1 (XLSX 28 kb)

**Supplementary Figure 1**. Loadings (eigenvectors coefficients) for markers (n = 9341 SNPs) on principal components 1 (PC1) and 2 (PC2). Each dot in the graph represents a marker, with its respective position shown on the x-axis, while the y-axis represents its contribution to the variation explained by each principal component (PC) . Mbp = Mega-base pair.
Supplementary file2 (TIFF 25312 kb)

**Supplementary Figure 2**. Proportion of advanced oat breeding lines belonging to the different chromosome rearrangement clusters according to nursery year. OTHER_V = varieties developed by other breeding programs, SDSU_V = Varieties developed by the SDSU Oat Breeding Program, ALL = all lines together were considered, while the other categories on the x-axis refer only to specific preliminary yield trials (PYTs) conducted in different years.
Supplementary file3 (TIFF 25312 kb)

**Supplementary Figure 3**. Allele profiles of progenies and their respective parents belonging to different clusters for chromosomal rearrangement regions. In the analyzed population, no progenies were derived from crosses between parents from Clusters 2 and 3 for the 7D inversion. The black lines indicate the boundaries (start and end) of the rearranged regions. The x-axis shows the SNPs, numbered sequentially according to their order and position on the respective chromosome, while the y-axis shows the parents (bottom two names) and the progenies (names above the parents).
Supplementary file4 (TIFF 36914 kb)

**Supplementary Figure 4**. Pairwise genetic distances in regions with chromosomal rearrangements by cluster classification. *** indicates a p-value less than 0.001, ** indicates a p-value less than 0.01, * indicates a p-value less than 0.05 and ‘ns’ indicates a p-value higher than 0.05.
Supplementary file5 (TIFF 37968 kb)

**Supplementary Figure 5**. Effect of chromosomal rearrangement clusters on grain yield (kg.ha-1), crown rust severity (%), heading date (Julian days), and plant height (centimeters). The values used were the BLUEs computed based on Equation 1. Difference in letters means a statistical difference between clusters (Šidák correction).
Supplementary file6 (TIFF 82265 kb)

**Supplementary Figure 6**. Phenotypic distribution of 1A/1C translocation clusters for grain yield (kg.ha-1), crown rust severity (%), heading date (Julian days), and plant height (centimeters) based on the nursery origin. Different letters indicate statistically significant differences between clusters based on Tukey’s post hoc test. Crown rust data were not collected in the 2021 year.
Supplementary file7 (TIFF 109687 kb)

**Supplementary Figure 7**. Phenotypic distribution of 3C inversion clusters for grain yield (kg.ha-1), crown rust severity (%), heading date (Julian days), and plant height (centimeters) based on the nursery origin. Different letters indicate statistically significant differences between clusters based on Tukey’s post hoc test. Crown rust data were not collected in the 2021 year.
Supplementary file8 (TIFF 109687 kb)

**Supplementary Figure 8**. Phenotypic distribution of 4C inversion clusters for grain yield (kg.ha-1), crown rust severity (%), heading date (Julian days), and plant height (centimeters) based on the nursery origin. Different letters indicate statistically significant differences between clusters based on Tukey’s post hoc test. Crown rust data were not collected in the 2021 year.
Supplementary file9 (TIFF 109687 kb)

**Supplementary Figure 9.** Phenotypic distribution of 7D inversion clusters for grain yield (kg.ha-1), crown rust severity (%), heading date (Julian days), and plant height (centimeters) based on the nursery origin. Different letters indicate statistically significant differences between clusters based on Tukey’s post hoc test. Crown rust data were not collected in the 2021 year
Supplementary file10 (TIFF 109687 kb)

## Data Availability

The genotyping dataset is available on T3/Oat (triticeaetoolbox.org).
